# Perceptions of dental professionals on the use of silver diamine fluoride with or without light-curing

**DOI:** 10.1186/s12903-024-05359-3

**Published:** 2024-12-31

**Authors:** Win Myat Phyo, Palinee Detsomboonrat

**Affiliations:** 1 International Graduate Program in Dental Public Health, Department of Community Dentistry, Faculty of Dentistry, Chulalongkorn University, Bangkok, Thailand; 2 Department of Community Dentistry, Faculty of Dentistry, Chulalongkorn University, 34 Henri-Dunant rd., Wangmai, Pathumwan, Bangkok, 10330 Bangkok Thailand

**Keywords:** Sliver diamine fluoride, Light-curing, Dental professionals, Perspective, Qualitative research

## Abstract

**Background:**

According to anecdotal reports, SDF’s ability to arrest caries can be enhanced by light-curing in a clinical setting. The purpose of the present study was to explore the dental professionals’ perceptions of using SDF and to understand the barriers and enabling factors to using SDF with and without light-curing.

**Methods:**

A qualitative study was conducted with dental professionals who had experience with using SDF with and without light-curing. A purposive heterogeneous and convenience sampling approach was applied to ensure the inclusion of participants with different employment and experience levels. Eighteen participants (dental students (undergraduate and postgraduate), dental personnel (practicing dentists and dental hygienists), and academics) with ages ranging from 22 to 53 years old participated in the study. The data were collected through a semi-structured open-ended questionnaire. Manual line-by-line coding and content analysis methods were used for data analysis.

**Results:**

Most participants indicated a preference for light-curing, citing perceived benefits, such as quicker treatment, convenience, better visibility, prevention of saliva contamination, and ensuring thorough SDF coverage. However, some participants voiced concerns, based either on existing practice guidelines or due to insufficient evidence regarding the efficacy of light-curing. Additionally, one participant proposed a hybrid approach based on tooth location, suggesting avoiding using light-curing on anterior teeth, while utilizing it on posterior teeth.

**Conclusion:**

The participants’ responses indicated that the use of SDF with and without light-curing each has its merits. Using SDF with light-curing emerged as the preferred method due to perceived benefits. e.g., quicker treatment, better moisture control. However, concerns regarding existing guidelines and the lack of robust evidence for its efficacy were also identified. This study highlights the need for additional research to address the knowledge gaps and provide stronger evidence to support the use of light-curing after SDF application.

**Supplementary Information:**

The online version contains supplementary material available at 10.1186/s12903-024-05359-3.

## Background

Untreated dental caries is a global public health concern. The 2019 Global Burden of Disease study found a higher number of prevalent cases of dental caries, with approximately 2.03 billion for permanent teeth and estimated 0.52 billion for primary teeth. Despite being largely preventable and controllable, more scientific evidence is needed to solidify the most effective methods for the prevention and control of dental caries [1]. The use of silver diamine fluoride (SDF) for managing dental caries has recently gained widespread attention. Dentists worldwide are increasingly using SDF because of its relative affordability and ease of implementation [2].

Empirical evidence has demonstrated SDF’s effectiveness in arresting dental caries in primary and permanent teeth. It has four major mechanisms of actions due to its ingredients. The silver component acts as an antimicrobial agent and impedes new biofilm formation, while the fluoride strengthens the tooth structure by preventing demineralization and promoting remineralization [3]. Evidence-based clinical practice guidelines on nonrestorative treatments for dental caries by the American Dental Association (ADA) recommend clinicians prioritize the use of SDF for cavitated dental caries on any coronal surface of primary and permanent teeth [4].

The American Academy of Pediatric Dentistry (AAPD) also recommends the use of SDF for managing dental caries in children, adolescents, and individuals with special health care needs with the goals of a dental home [5]. Moreover, it has recently been included in the lists of essential medicines by the World Health Organization (WHO) [6] and endorsed for managing coronal and root caries by the Ministry of Public Health in Thailand [7]. Moreover, the emergence of the COVID-19 pandemic has raised concerns regarding the spread of the coronavirus via aerosols produced during dental procedures. Utilizing SDF presents a favorable choice for managing dental caries due to its non-production of aerosols and minimal risk of cross-infection [8].

According to the reported practical recommendation by AAPD, the optimum time for SDF application should be one minute, using a gentle flow of compressed air until the liquid is dry. However, the duration of application should be reduced in very young children and challenging patients. It may be challenging to manage shorter application times, requiring close monitoring during the post-operative and follow-up visits to assess the effectiveness and contemplate the need for re-application [5]. Crystal and Niederman hypothesized that dental caries in primary anterior teeth, when treated with SDF, exhibited a higher rate of caries arrest compared with posterior teeth due to the easier cleaning of anterior teeth and their greater exposure to natural light [9]. In Fung et al., a higher caries arrest rate was found in anterior teeth compared with posterior teeth after SDF application [10]. Therefore, light may play a role in arresting dental caries with SDF because natural light may lead to reduced SDF soaking time and greater likelihood of silver precipitation [9].

Light-emitting diode (LED) curing lights have emerged as the prevailing choice for curing restorative materials in dentistry and the use of LED curing lights is widespread across dental practices. Based on laboratory studies, SDF with light-curing technique has a range of positive outcomes, including increased microhardness, higher mineral density, and more precipitation of silver ions in infected dentine and maintaining its antibacterial property [3, 11–15]. One of the possible mechanisms is that light-curing may enhance the precipitation of silver ions within the dentine layer of carious lesions and increase the surface hardness of the carious lesions [3]. Therefore, it could be hypothesized that the addition of light-curing following SDF application might enhance the efficacy of SDF in arresting dental caries.

Although anecdotal evidence suggested that many clinicians routinely utilized light-curing on SDF after its application; there was no clinical evidence regarding the effect of light-curing on SDF after its application in a clinical setting to arrest dental caries [9]. Therefore, there is a need to characterize the contemporary barriers and enabling factors for using SDF either with or without light-curing. However, there has been no qualitative study investigating the perspective of dental professionals on the use SDF, either with or without light-curing technique. Therefore, the purpose of the current study was to explore the views of dental professionals on using SDF either with or without light-curing in Thailand.

## Methods

### Study design

This study had a qualitative exploratory design focusing on the dental professionals’ perspectives of using SDF with or without light-curing. This study had been performed in accordance with the Declaration of Helsinki and was approved by the Human Research Ethics Committee (HREC-DCU 2023 − 137) of the Faculty of Dentistry, Chulalongkorn University, Bangkok, Thailand.

### Participant recruitment

A purposive heterogeneous and convenience sampling approach was applied to ensure the inclusion of participants with different employment and experience levels. The participants were gathered through social media, private dental practices, snowball sampling, and the personal connections of the research team. The participants were approached via phone, email, and the LINE application. The eligibility criteria comprised dental professionals who had experience with using SDF with and without light-curing. These consisted of dental students (undergraduate and postgraduate), dental personnel (practicing dentists and dental hygienists) and academics, of any age. Before participating in the study, the participants had the opportunity to ask any questions and ensure they were willing to participate, and consent was explained and obtained. The recruitment process stopped once data saturation was achieved [16].

### Data collection and analysis

A semi-structured open-ended questionnaire was electronically responded to by 18 participants, categorized into 3 groups of dental professionals: dental students (undergraduate and postgraduate), dental personnel (practicing dentists and dental hygienists), and academics. The questionnaire was developed based on SDF availability, barriers, enabling factors, comparative experience and additional comments/suggestions (See Additional file 1). Data were collected during February-April 2024. To provide the participants with a summary of the study’s findings upon its conclusion, their contact information was collected if they wished to receive this.

The responses were recorded in text form and fully transcribed from Thai language into English (WMP and PD). No qualitative data analysis computer software package was used because the responses were brief, and the volume of text data was not large. Two researchers (WMP and PD) read the transcripts several times to become familiar with them. The data were analyzed by the content analysis method [17] and any disagreements between the 2 researchers (WMP and PD) were resolved through discussion. The qualitative research report was developed through the integration of the findings. To improve trustworthiness, these findings underwent member checking by 5 participants [18].

## Results

### General characteristics

Eighteen participants (3 males and 15 females) with ages ranging from 22 to 53 years old participated in the study (Table 1). No participants refused to participate or withdrew consent. Many participants were very familiar with SDF (38.9%) and worked in both private and government sectors (44%).

**Table 1:** Demographics of the participants in this study

Response	Age (year)	Sex	Role	Specialty	Year of practice	How much familiar with using SDF
1	20–30	Female	Postgraduate Student	Pediatric Dentistry	≤ 5	Very familiar
2	20–30	Male	Postgraduate Student	Dental Public Health	≤ 5	Slightly familiar
3	20–30	Female	Dentist	Pediatric Dentistry	6–10	Very familiar
4	31–40	Female	Academic	Pediatric Dentistry	> 10	Very familiar
5	41–50	Female	Academic	Pediatric Dentistry	> 10	Somewhat familiar
6	50+	Female	Academic	Dental Public Health	> 10	Somewhat familiar
7	20–30	Female	Postgraduate Student	Pediatric Dentistry	≤ 5	Very familiar
8	31–40	Female	Dental Hygienist	Dental Hygienist	> 10	Somewhat familiar
9	20–30	Female	Undergraduate Student	Undergraduate Student	≤ 5	Somewhat familiar
10	20–30	Male	Dental Hygienist	Dental Hygienist	≤ 5	Slightly familiar
11	20–30	Female	Undergraduate Student	Undergraduate Student	≤ 5	Slightly familiar
12	20–30	Female	Undergraduate Student	Undergraduate Student	≤ 5	Slightly familiar
13	50+	Female	Academic	Pediatric Dentistry	> 10	Very familiar
14	31–40	Female	Dentist	Pediatric Dentistry	6–10	Very familiar
15	31–40	Female	Academic	Geriatric Dentistry	6–10	Somewhat familiar
16	41–50	Female	Dentist	Operative Dentistry	> 10	Slightly familiar
17	20–30	Male	Undergraduate Student	Undergraduate Student	≤ 5	Slightly familiar
18	31–40	Female	Academic	Pediatric Dentistry	6–10	Very familiar

### Qualitative findings

#### Product availability

The participants in this study identified the limited availability of SDF in Thailand as the main barrier for using SDF. Most of the participants expressed interest in using SDF with light-curing because the light-curing unit was already present in daily dental practice. There was no policy that either supported or hindered the use of SDF with light-curing. In contrast, other participants expressed a need for results from evidence-based research before performing SDF with light-curing. They also indicated that they required additional information and training regarding the precise usage instructions, particularly the duration for applying SDF and the use of the light-curing unit during each step of the treatment process.

### Barriers to, and enabling factors for using SDF either with or without light-curing

**Fig. 1:**
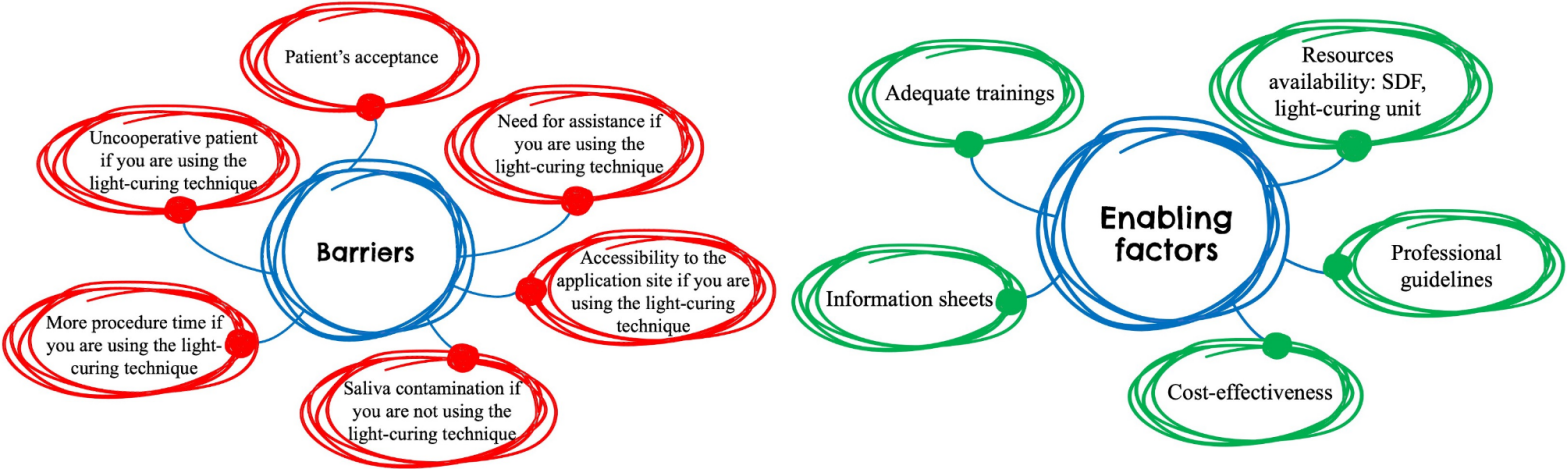
Summary of barriers and enabling factors for the use of SDF either with or without light-curing

The findings of this study identified several barriers and enabling factors influencing the use of SDF with or without light-curing. To provide a clearer and more accessible summary, a visual representation of these key outcomes is presented (Fig. 1).

### Barriers

Patient’s cooperation and acceptance, procedure time, saliva contamination, accessibility to the application site and requirement for assistance were common barriers among participants to using SDF either with or without light-curing.

*When we are planning to use light-curing*, *we may need someone to help*, *especially with young uncooperative patients*, *and sometimes it may be difficult to access the application site particularly on the buccal surfaces of posterior teeth.* ***(Response 07 and 16)***.

*After applying SDF*, *if not followed by light-curing*, *it may need more time for moisture isolation to prevent saliva contamination.* ***(Response 10)***.

### Enabling factors

In addition to identifying potential obstacles to the use of SDF, both with and without light-curing, the factors that could facilitate its application were also explored. It was proposed that creating new training courses or professional development events to establish professional guidelines could help overcome the shortage of training opportunities and potentially boost its practical application. To aid in introducing SDF to parents, it was suggested to provide an information sheet. This sheet should detail the benefits, drawbacks, and anticipated results, along with photographs illustrating arrested carious lesions.

*It would be nice to have pictures showing how the teeth turn black after treatment with SDF only and SDF with light-curing*, *and possible side effects.* ***(Response 01 and 03)***.

Most of the participants acknowledged that there was no difference in term of the cost-effectiveness of using SDF with light-curing compared with using it without light-curing. However, a few participants suggested to use light-curing after SDF application if there was a light-curing unit in their practice.

*Using light-curing will be worthwhile. The carious lesions will become hard and darker after being exposed to light-curing. If you don’t use light-curing after SDF application*, *there may be contamination with saliva*, *and you may have to apply it again if you could not control the saliva well.* ***(Response 10)***.

### Comparative views of using SDF with light-curing to without light-curing

Only a few participants raised points about no difference in terms of ease of application between using SDF with and without light-curing. The majority of participants expressed that SDF with light-curing was easy to perform. However, in terms of the longevity or durability of caries arrest, most participants responded that there was no difference between the two methods.

*Using SDF with light-curing is easier because it helps the patient to see dark areas more clearly as soon as after application and it is easy to perform with young children.* ***(Response 08 and 09)***.

*I haven’t seen any difference in the longevity or durability of caries arrest between the two methods. It may depend on the patient’s oral hygiene.* ***(Response 05)***.

Most participants felt that the majority of parents were supportive of the treatment mainly due to the dentists’ endorsement, as they trusted the dentists’ advice. Most participants raised points about the similarities in parents’/patients’ acceptance between the two methods. Two participants, however, stated using SDF with light-curing would make parents/patients more satisfied.

*If light-curing is applied*, *parents and patients can see black as shown in the picture before starting the treatment. Most parents and patients may not encounter any problems when they come back for follow-up. When light-curing is not used after SDF application*, *the black is still not clearly visible after its application. When they are coming back for follow-up*, *some may feel dissatisfied with the treatment results.* ***(Response 05 and 13)***.

The majority of participants expressed a preference for using SDF with light-curing compared with the conventional application of SDF. However, a few participants voiced concerns about the lack of strong evidence.

*I like using SDF with light-curing because it saves time and ensures that SDF covers the desired area.* ***(Response 18)***.

*I prefer to use SDF without light-curing because there is no guideline/strong evidence of using SDF with light-curing.* ***(Response 02)***.

However, one participant responded with a hybrid approach based on tooth position, suggesting the avoidance of using light-curing on anterior teeth while utilizing it on posterior teeth.

*For anterior teeth*, *I use SDF without light-curing due to the possibility of natural light exposure*, *but I use SDF with light-curing for posterior teeth.* ***(Response 14)***.

### Additional comments/experiences

In terms of clinical/practical considerations, ensuring cleaning and removing food debris and moisture isolation before applying SDF was mentioned as crucial for better performance.

*Isolating the teeth to make them as dry as possible improves the performance of SDF.* ***(Response 03)***.

*Cleaning and removing food debris first before applying SDF helps you to get better results.* ***(Response 11)***.

There were benefits and challenges in some specific contexts. Using SDF benefitted patients with special health care needs or patients who were on the waiting list for full mouth rehabilitation (FMR) under general anesthesia (GA) [19]. However, there were also challenges in patients whose teeth had been applied with SDF before FMR under GA. There was a question of whether the outer dark layer of carious lesion should be removed to check beneath the surface, along with concerns about possible pulp exposure and limited visibility.

*When using SDF to arrest caries in patients awaiting full mouth rehabilitation under general anesthesia*, *the subsequent procedure can be challenging. During FMR under GA*, *treatment tends to be more aggressive than in a typical dental setting to avoid the need for follow-up procedures with uncooperative patients. When encountering a black lesion*, *which indicates an arrested cavity*, *there’s concern about whether the cavity beneath is also arrested. This raises the question of whether the outer layer should be removed to check the condition inside. Additionally*, *there’s uncertainty about the risk of exposing the pulp and the difficulty of seeing through the black*, *hardened surface.* ***(Response 04)***.

## Discussion

This research explored the perceptions of dental professionals on using SDF with and without light-curing and the results demonstrated that the perceptions were influenced by multiple factors. To the best of our knowledge, this is the first study to explore the perspectives of dental professionals on SDF application for caries arrest, with a specific focus on their experiences with and without light-curing, using a qualitative approach. These findings highlight the barriers and enabling factors associated with each approach, providing insightful information that can be used to guide future research and serve as a starting point for implementation planning aimed at enhancing SDF-specific knowledge among clinicians. Qualitative research offers a deeper understanding of attitudes and the ability to identify difficulties a priori that are not achievable with quantitative research [20].

Selecting between SDF with and without light-curing is influenced by individual preferences and the specific context of their practice environment. Participants’ preference leaned toward the novel technique, SDF with light-curing, compared with the conventional application method. This preference stemmed from perceived advantages, including increased convenience, reduced treatment duration, and the generation of immediate, visible outcomes. These perceived benefits were hypothesized to contribute to improved patient management (e.g., expectation setting), reduced risk of saliva contamination, and potentially enhanced SDF coverage. However, a minority of participants voiced concerns regarding the current lack of definitive guidelines for using SDF with light-curing and the need for more robust evidence on its efficacy.

This study identified training opportunities as crucial for overcoming the perceived barriers to SDF implementation. The participants suggested workshops or informational resources (fact sheets or videos) to familiarize themselves and their patients with SDF application with and without light-curing. Patient education materials were seen as instrumental in empowering parents/patients to participate in treatment decisions, particularly regarding the choice between the two methods.

Furthermore, the participants expressed enthusiasm for the potential application of light-cured SDF in managing caries in specific patient populations. They envisioned its use as a transitional or definitive treatment, particularly for very young children or patients with limited treatment tolerance due to medical or psychological factors (e.g., frail elderly, individuals with disabilities, dental phobias). However, the participants emphasized the need for further clinical studies to validate the efficacy of SDF with light-curing in arresting caries progression.

Our findings resonate with the existing literature on clinical decision-making, which highlights the influence of factors, such as guidelines, parental/patients’ preferences, colleague support, and clinician beliefs [21–23]. Furthermore, our results align with research on caries treatment decision-making, which emphasizes the interplay of parental and patients’ preferences, evidence-based practices, and dental professionals’ preference [23, 24]. The present study corroborates findings from prior trials, suggesting high interest in the novel technique of applying SDF with light-curing [3, 25].

Although this study benefits from the inclusion of participants from diverse dental backgrounds, offering a broad perspective on attitudes and reactions, the sample size might be considered modest. However, this is not uncommon for qualitative research endeavors [26]. Future studies employing a larger and more geographically diverse sample of dental professionals with a wider range of clinical experiences are recommended to enhance the generalizability of our findings.

It is important to acknowledge the potential limitations in the interpretation of the results. The research team had a working, professional relationship with the participants, which can introduce potential bias, particularly when researchers, as being insiders, develop a sense of shared understanding with participants [23, 27, 28]. This may lead to overlooking alternative interpretations of participant statements, potentially hindering the identification of new research avenues [23]. Moreover, nearly half (44%) of the participants possessed ≤ 5 years of professional experience. Therefore, their perspectives may evolve as their clinical practice matures. Despite their general experience, they may possess more familiarity with SDF compared to practitioners who graduated 15 years ago but have not consistently used SDF in their practice. In addition, the context of this study primarily reflects the perspectives of female dental professionals, while the viewpoints of their male counterparts are less represented. The majority of Thai dentists are female, accounting for 66.2%, while males make up 33.8%. We believe that the perspectives of female dentists accurately may reflect and represent the demographics of dental practitioners in Thailand [29].

## Conclusions

Based on the findings of this study, dental professionals believe that the use of SDF with and without light-curing each has its merits. Using SDF with light-curing emerged as the preferred method due to perceived benefits including quicker treatment, and better moisture control. However, concerns regarding existing guidelines and the lack of robust evidence for its efficacy were also identified. To conclusively establish the long-term clinical effects of SDF with light-curing relative to the conventional application method, further investigation is needed. Moreover, research examining the cost-effectiveness of these two methods and their influence on patients’/parents’ adherence would be highly beneficial. Gaining insight into the dental professionals’ perspectives on the respective barriers and enabling factors of each approach can aid clinicians in determining the most appropriate method for their practice, thereby enhancing patient care outcomes.

## Electronic supplementary material

Below is the link to the electronic supplementary material.

Supplementary Material 1

## Data Availability

The datasets used and/or analysed during the current study are available from the corresponding author on reasonable request.

## Abbreviations

SDF: Silver diamine fluoride
ADA: American Dental Association
AAPD: American Academy of Pediatric Dentistry
WHO: World Health Organization
LED: Light-emitting diode
FMR: Full mouth rehabilitation
GA: General anesthesia
