# Supplementary material for: Perceptions of dental professionals on the use of silver diamine fluoride with or without light-curing
Source: BMC Oral Health. 2024 Dec 31;24:1578. doi: 10.1186/s12903-024-05359-3 (PMC11689526; doi:10.1186/s12903-024-05359-3)
Supplement: Supplementary file 1 — Supplementary Material 1 [file 12903_2024_5359_MOESM1_ESM.docx]

**QUESTIONNAIRE**

Response ID …………

Thank you for your participation in our research project.

**Section A**: Demographic information

1. **What is your age and gender?**
2. **What is your specialty and current position?**
3. **In what sector are you currently working?**
4. **How many years of clinical experience do you have?**
5. **How familiar are you with SDF?**

**Section B**: Questionnaire Guide for Qualitative Study

1. How is SDF avaialble in your practice?
2. **In your experience, what are the biggest challenges and advantages you have encountered when using SDF, both with and without light curing?**
3. **Based on your practical experience, can you share any insights (in terms of ease of application, effectiveness, parental acceptance etc.,) on using SDF with or without light curing?**
4. **Is there anything else you would like to share about your experience using SDF, regardless of the curing method?**
